# Quantification of carious pathogens in the interdental microbiota of young caries-free adults

**DOI:** 10.1371/journal.pone.0185804

**Published:** 2017-10-10

**Authors:** Denis Bourgeois, Alexandra David, Camille Inquimbert, Paul Tramini, Nicolas Molinari, Florence Carrouel

**Affiliations:** 1 Laboratory "Systemic Health Care" EA4129, University Lyon 1, Lyon, France; 2 Department of Prevention and Public Health, Faculty of Dentistry, University Lyon 1, Lyon, France; 3 Department of Dental Public Health, University of Montpellier, Montpellier, France; 4 Service DIM, CHU de Montpellier, UMR 5149 IMAG, University of Montpellier, Montpellier, France; 5 Department Basic and Clinical Biological Sciences, Faculty of Dentistry, University Lyon 1, Lyon, France; Universidad Nacional de la Plata, ARGENTINA

## Abstract

**Background:**

The majority of caries lesions in adults occur on the proximal tooth surfaces of the posterior teeth. A comprehensive study of the composition of the oral microbiota is fundamental for a better understanding of the etiology of interdental caries.

**Methods:**

Twenty-five caries-free subjects (20–35 years old) were enrolled in the study. The interdental biofilm of four interdental sites were collected. The real-time polymerase chain reaction (PCR) methodology were used to quantify (i) the following bacteria: *Streptococcus spp*., *Streptococcus mutans*, *Lactobacillus spp*., *Enterococcus spp*., and *Enterococcus faecalis;* (ii) the fungus *Candida albicans;* and (iii) *total bacteria*.

**Results:**

*Streptococcus* spp. was the most abundant species, followed by *Lactobacillus* spp. and *Enterococcus* spp. *Streptococcus* spp. and *Lactobacillus* spp. were detected at all tested sites and *Enterococcus* spp. at 99% of sites. *S*. *mutans* was detected at only 28% of the tested sites and *C*. *albicans* was detected at 11% of sites. *E*. *faecalis* was never detected. In 54.5% of the biofilm inhabited by *C*. *albicans*, *S*. *mutans* was present. Moreover, 28% of the ID sites co-expressed *S*. *mutans* and *Lactobacillus* spp. The studied pathogens were organized into two correlated groups of species. Strikingly, the fungus *C*. *albicans* and the bacteria *Enterococcus* spp. cluster together, whereas *Streptococcus* spp., *S*. *mutans* and *Lactobacillus* spp. form one distinct cluster.

**Conclusion:**

The interdental biofilm of young caries-free adults is comprised of pathogens that are able to induce interproximal caries. That several of these pathogens are implicated in heart disease or other systemic diseases is an argument for the disruption of interdental biofilms using daily oral hygiene.

## Introduction

The 2010 Global Burden of Disease Study found that oral conditions affected 3.9 billion people worldwide and that the estimation of untreated caries of permanent teeth was 2.4 billion [1, 2]. Dental caries is a multifactorial, chronic bacterial disease that may result in cavity formation in the enamel, dentine and cementum [3].

The incidence of untreated caries predominates below the age of 35 and decreases with increasing age, although it remains a significant problem in the upper age categories [4]. The majority of caries lesions in adolescents and adults occur on the proximal tooth surfaces of the posterior teeth [5, 6, 7].

Many distinct habitats may be identified on individual teeth, with each habitat containing a unique biofilm community [8]. Tooth habitats favorable for harboring pathogenic biofilm include the smooth enamel surfaces immediately gingival to the proximal contacts and in the gingival third of the facial and lingual surfaces of the clinical crown [9]. These areas are protected physically and are relatively free from the effects of mastication, tongue movement, and salivary flow [9]. Local gingival changes in this area will lead to a protected surface for biofilm accumulation [10]. The relationship between gingivitis and caries on the proximal surface is narrow [11].

More importantly, the microbial structure varies with ageing. In addition, only a few taxa are present across the entire population, indicating that a core oral microbiome should be defined based on age and oral niche [12]. The types and numbers of organisms composing the proximal surface biofilm community vary [13]. The mesial surface of a molar may be carious and have a biofilm dominated by large populations of *Streptococcus mutans* and lactobacilli, whereas the distal surface may lack these organisms and be caries-free [13]. The intra- and inter-individual progression of proximal caries fluctuates, indicating different cariogenic conditions [14].

The literature on interdental (ID) supragingival microbial profiles applied to caries lesions is extremely limited. Currently, no studies have addressed the ID biofilm of caries-free adults. It remains unclear which microorganisms positively or negatively impact patients with regards to clinical considerations [15, 16].

The goal of this study is to describe the interproximal microbiota in caries-free young adults. Thus, a quantitative detection method using real-time polymerase chain reaction (PCR) was employed to quantify 6 major cariogenic pathogens, including (i) the bacteria: *Streptococcus* spp. (*S*spp), *Streptococcus mutans* (*S*. *mutans*, *Sm*), *Lactobacillus* spp. (*L*spp), *Enterococcus* spp. (*E*spp), and *Enterococcus faecalis* (*E*. *faecalis*, *Ef*); and (ii) the fungus *Candida albicans* (*C*. *albicans*, *Ca*).

The results of this research can be used to considerably improve the dental condition of adolescents and young adults. Standard dental therapy does not yet include any microbiological based approach into its armamentarium. The results can be used to make decisions with respect to molecular analyses for new policies covering the provision of services instituting new procedures (e.g., micro-invasive treatment of proximal caries lesions), practices and interventions (e.g., non-invasive professional treatment) or to provide advice for prevention (e.g., an interdental brush (IDB)) related to dental health care delivery.

## Materials and methods

**Fig 1:**
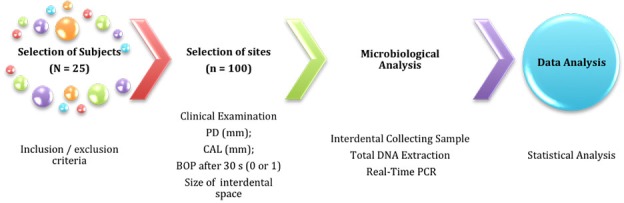
Workflow of the experiment.

The workflow of this research is detailed in Fig 1.

### Subject population

Twenty-five Caucasian subjects diagnosed as caries-free were recruited between January and April 2015 from a pool of first-time volunteers who were referred to the Department of Public Health of the Faculty of Oral Medicine at the University of Lyon (UCBL), France. Written informed consent was obtained from all enrolled individuals in accordance with the Declaration of Helsinki. The study protocol was reviewed and approved by the Local Ethics Committee and by the National Commission of Informatics and Liberties, France.

The inclusion criteria were (i) 20–35 years old (male or female), (ii) good general health, not pregnant or breastfeeding and on contraceptive therapy, (iii) good oral hygiene, (iv) good diet (Healthy Eating Index score greater than 80), (iv) no health conditions that required antibiotic prophylaxis before interproximal probing, (v) no oral diseases (such as dental caries, periodontal disease, periapical disease, oral mucosal disease, or severe halitosis), (vi) tooth brushing at least twice per day, (vii) no experience with interdental cleaning—interdental brushing or dental flossing, (viii) no intake of systemic antimicrobials during the previous 6 months, (ix) no use of chlorhexidine or over-the-counter mouthwash, (x) no implants or orthodontic appliances, (xi) no previous periodontal illness or treatment, (xii) the presence of at least 24 natural teeth, (xiii) the presence of 4 premolar-molar pairs, (xiv) non-smokers, and (xv) a willingness to return 3 weeks after the clinical investigation for microbiological tests.

The clinical inclusion criteria for each premolar-molar interdental site were (i) accessibility of the interdental space for the 4 sites (15–16, 25–26, 35–36, and 45–46, according to the FDI’s two-digit notation system [17]) by the interdental brush in each subject, (ii) no interproximal caries or dental or prosthetic restorations, (iii) no interdental diastema, (iv) no clinical signs of inflammation, such as redness, swelling, or bleeding on probing (BOP) after 30 s, (v) no pocket depth (PD) or PD ≤ 3 mm or clinical attachment loss (CAL) > 3 mm, and (iv) the subjects were judged to be free of gingivitis or periodontitis.

The exclusion criteria were (i) teeth missing due to periodontal reasons, (ii) having any other concomitant systemic disorder, (iii) having diseases affecting the immune system, (iv) receiving medication, such as anti-platelet or anti-coagulant agents, (v) having a professional prophylaxis 4 weeks prior to the baseline examination, (vi) having a history of periodontal disease or treatment, and (vii) subjects undergoing a course of dental or orthodontic treatment.

### Classification of subjects as caries-free

The dental health status of individuals was determined by measuring the Decayed, Missing, and Filling Teeth (DMFT) index. This index is recognized in epidemiology for assessing dental caries prevalence and indicates the necessary treatments. Moreover, the DMFT index was recorded to measure the severity of each subject's dental caries according to the criteria from the World Health Organization 4th-edition publication of "Oral Health Surveys, Basic Method" [18].

### Clinical examination

Standardized clinical monitoring was performed three weeks before microbiological monitoring. The subjects were submitted to a medical/dental anamnesis, and information regarding subject age, gender and smoking status was obtained. A trained and calibrated professional dentist performed the clinical examination. Clinical assessments of the interdental spaces were performed using an IAP Curaprox colorimetric probe (Curaden, Kriens, Switzerland), and the diameters of all the interdental spaces of 4 teeth were registered (premolar-molar). At the end of the examination visit, the participants were instructed to brush their teeth 3 hours before the sampling visit and not to drink, eat or practice oral hygiene during this period.

### Interdental sample collection

For all subjects, the same four interdental sites (15–16, 25–26, 35–36, and 45–46) were assessed (total of 100 sites). The appropriate CPS prime interdental brushes (Curaden, Kriens, Switzerland) were selected based on the clinical assessment of the interdental spaces [19]. Each previously selected tooth was isolated with sterile cotton rolls and the interdental biofilm was removed with a sterile, calibrated interdental brush. For each sample, the IDBs were placed in 1.5 mL sterile microcentrifuge tubes and stored at 4°C until the DNA was extracted one hour later.

### Microbiological analysis

#### Total deoxyribonucleic acid (DNA) extraction

Total DNA was isolated from the interdental brushes using the QIAcube^®^ HT Plasticware and Cador^®^ Pathogen 96 QIAcube^®^ HT Kit (Qiagen, Hilden, Germany) according to the manufacturer’s guidelines. The elution volume used in this study was 150 μL. DNA quality and quantities were measured using an ultraviolet spectrophotometer. The DNA sample was considered pure if the A260/A280 ratio was in the range of 1.8–2 and the A260/A230 ratio was in the range of 2–2.2.

#### Quantitative real-time PCR assays

To quantify the total bacterial load (TB) and that of 6 pathogens (*Streptococcus* spp., *S*. *mutans*, *Lactobacillus* spp., *Enterococcus* spp., *E*. *faecalis*, and *C*. *albicans)* present in the biofilm interdental samples, qPCR was undertaken using universal primers for the 16S rRNA genes and species-specific primer sets. Each sample was analyzed in triplicate.

The *Ca* strain (DSM No. 6659), *E*spp strain (*Enterococcus faecalis DSM No*. *24916*), *Ef* strain (*DSM No*. *24916*), *L*spp strain (*Lactobacillus casei CIP No*. *102237*), *S*. *mutans* strain (*DSM No*. *20523*), and *S*spp strain (*S*. *mitis* DSM No. 12643) were obtained from DSMZ (Germany), the CIP Collection of the Institut Pasteur or from the BCMM/LMG Bacteria Collection and provided by Institut Clinident SAS (Aix en Provence, France).

The pathogenic strains were cultivated on the appropriate selective media. The total number of cells (number of colony forming units) was enumerated three times using a Neubauer chamber. Serial dilutions ranging from 10xE+2 to 10xE+12 cells were utilized, and each of these dilutions was enumerated in duplicate. The DNA from each of these dilutions was extracted. A standard curve for each pathogen was generated as a plot between the crossing point (cycle number) and the initial cell count. The TB standard curve was made from *Escherichia coli* as described by Ott and colleagues [20]. The limit of quantification (LOQ) of the method for each pathogen is summarized in Table 1.

**Table 1:** Species-specific and ubiquitous real-time PCR primers for 6 pathogens, the annealing temperature, and the limit of quantification.

Target	Primer pairs (5'-3')	References	Annealing temp (°C)	LOQ (E+02)
TB	CCATGAAGTCGGAATCGCTAGT GCTTGACGGGCGTGTG	[21]	66	200
*Ca*	ACTTCTGTAAGAGTGCTGGTTC TGTCGTAATCAAACTCGGTAGC	[22]	54	4
*E*spp	TACTGACAAACCATTCATGATG AACTTCGTCACCAACGCGAAC	[23]	55	5
*Ef*	CCGAGTGCTTGCACTCAATTGG CTCTTATGCCATGCGGCATAAAC	[24]	54	5
*L*spp	TGGAAACAGRTGCTAATACCG GTCCATTGTGGAAGATTCCC	[25]	62	10
*S*. *mutans*	GCCTACAGCTCAGAGATGCTATTCT GCCATACACCACTCATGAATTGA	[26]	66	8
*Streptococcus* spp.	AGAGTTTGATCCTGGCTCAG GTACCGTCACAGTATGAACTTTCC	[23]	66	10

LOQ: Limit of quantification; TB: Total bacterial count.

Simplex quantitative real-time PCR assays were performed in a 10 μL reaction composed of 1× SYBR^®^ Premix Ex Taq^TM^ Tli RNaseH Plus (TaKaRa, Shiga, Japan), 2 μL of the extracted DNA and 1 μM of each primer. The bacterial primers used are derived from previously published ribosomal 16S sequences and have been adapted to the real-time PCR conditions (Table 1). *Candida albicans* primers used in this study are derived from ribosomal 18S sequence. These PCR primers were manufactured by Metabion International AG (Planegg, Germany). For each pathogen, a positive and a negative control with sterile distilled water were included throughout the procedures.

The assays were performed on the Rotor-Gene^®^ Q thermal cycling system (Qiagen, Hilden, Germany) with the following program: 95°C for 30 s, followed by 40 cycles of 10 s at 95°C, 10 s at the appropriate annealing temperature (Table 1), and 35 s at 72°C. For the total bacterial load and that of all species, a final melting curve analysis (70°C to 95°C in 1°C steps at 5 s increments) was performed. Fluorescence signals were measured every cycle at the end of the extension step and continuously during the melting curve analysis. The resulting data were analyzed using Rotor-Gene^®^ Q Series software (Qiagen, Hilden, Germany).

### Statistical analysis

The statistical analysis consisted of three main steps: producing descriptive summaries of the data, modeling the data using a mixed (linear) model and assessing the correlations between bacterial abundances. Prior to these steps, we transformed the original count data to handle missing data points; that is, the measurements that fell under the quantification threshold (limit of quantification, LOQ) of the quantitative real-time PCR device. The missing values for a given species were replaced by half of the corresponding quantification thresholds given in Table 1. We performed simulations to ensure that this simple strategy provided a reasonable estimation of the mean and standard deviation of the original count distribution. To test for potential effects of sex, age, interdental space and the location of each site, we used a mixed linear model for the count abundance of each species at a measured site. This model includes two categorical variables as fixed effects (sex and mouth location), two numerical variables as fixed effects (age and interdental space) and one categorical variable as a random effect (subject). This random effect was introduced for a subject to model the correlation between the four sites of a given subject. Each coefficient in the regression was tested against the null hypothesis, which indicated that the coefficient is zero using a likelihood ratio test, and we reported that p-values less than 0.05 were evidence against the null hypothesis. To perform the correlation analysis, we used the residuals of the model described above to avoid over-estimating the inter-site correlation (sites from the same patient are positively correlated, and we observed that fixed effects can also induce a correlation among sites). The trees associated to the correlation plot were obtained by hierarchical clustering with complete linkage.

All statistical analyses and associated plots were performed using the R environment (R Core Team, 2015), specifically the lme4 package [27], to estimate the mixed model.

## Results

### Age, sex, and clinical characteristics of the study group

The sample group was composed of 15 males and 10 females 20 to 35 years of age with a mean body mass index of 22.7 (Table 2). Clinically, less than 10% of sites presented BOP after 30 s and/or overt gingival redness. No PD or PD ≤ 3 mm or CAL > 3 mm were observed. The subjects were characterized by a DMFT index of zero. The mean number of teeth was 28.9 ± 1.2. Missing teeth were due to absence of the third molars (97%) and orthodontic extractions (3%). A total of 60% of interdental spaces had a diameter less than 0.7 mm.

**Table 2:** Age, sex, and characteristics of the full mouth of the study group. The values are the mean ± standard deviation, and the numbers of subjects are indicated.

**Subjects**	
Age (years)	26.8 ± 4.6
Sex	
Male	15
Female	15 10
Body mass index	15 22.7 ± 1.8
**Mouth**	
Teeth	28.9 ± 1.2
Interdental space diameter (%)	
0.6 mm	5
0.7 mm	55
0.8 mm	25
0.9 mm	8
1.1 mm	7
Bleeding on probing (%)	0.16 ± 0.08
Plaque index	0.24 ± 0.52

### Individual pathogen count

**Fig 2:**
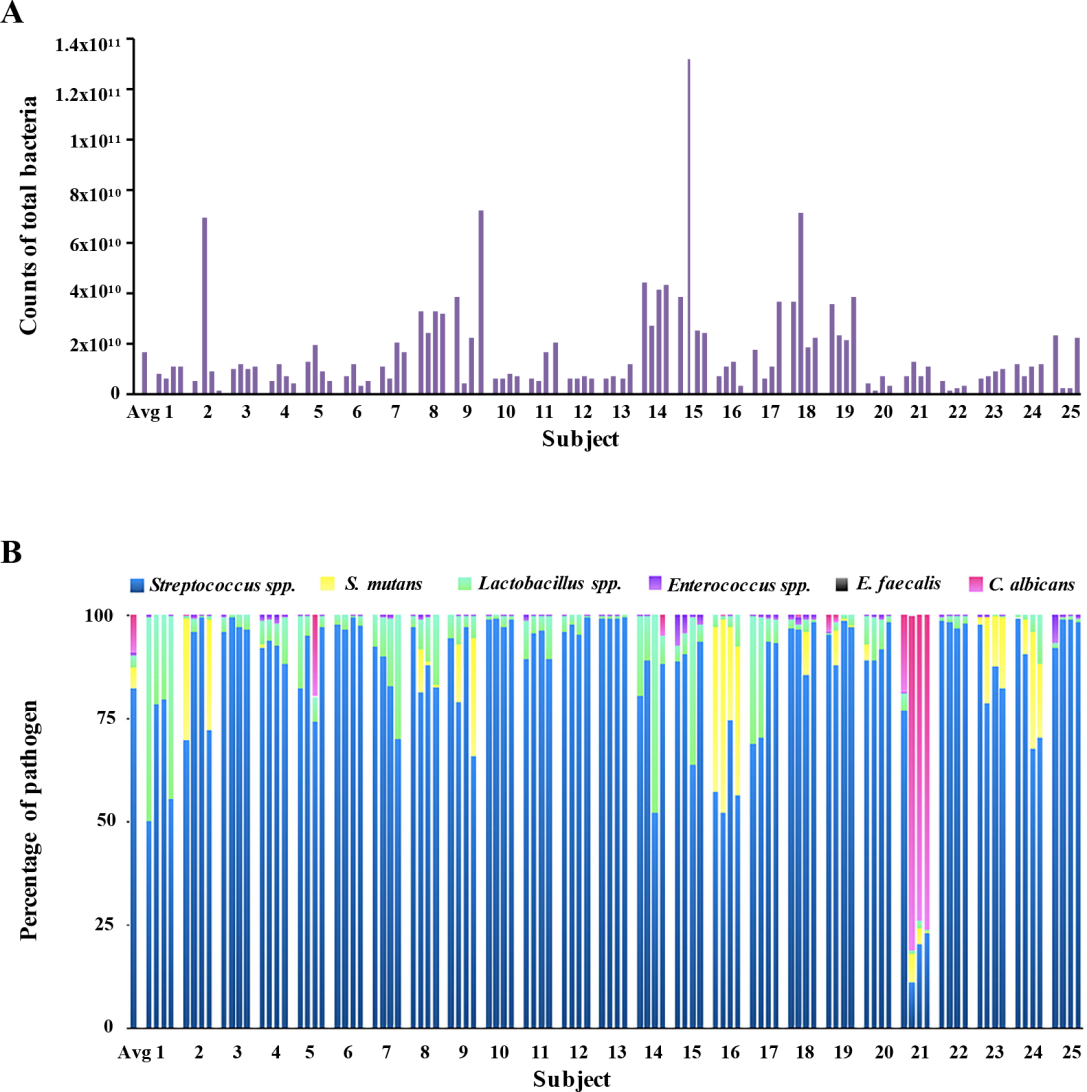
Abundance of pathogens among the subjects. A. Counts of total bacteria among the subjects. The first bar displays the average proportion of total bacteria in the population. The other bars display the average proportion of each pathogen in one site. Each subject corresponds to a group of four stacked bars (one for each measured site). B. Relative abundance of pathogens among the subjects. Percentage of pathogen = Counts of the pathogen / Counts of the 6 pathogens. The first bar displays the average proportion of each pathogen in the population. The other bars display the average proportion of each pathogen in one site. Each subject corresponds to a group of four stacked bars (one for each measured site). Avg: Average.

The count for the total of bacteria by subject is presented in Fig 2A and S1 Table. The proportion of the 6-evaluated species in the samples is described in Fig 2B and the frequency in Table 3. Variations between the subjects and the sites in the carriage of certain bacteria were observed. Subject 21 had high levels of *C*. *albicans*, whereas certain other subjects carried *S*. *mutans*, including subjects 2, 8, 9, 16, 23 and 24. *Streptococcus* spp. and *Lactobacillus* spp. were detected (number of bacteria > LOQ) at all tested sites and *Enterococcus* spp. at 99% of sites while *S*. *mutans* was detected at only 28% of the tested sites. *E*. *faecalis* was never detected. In 11% of sites, *C*. *albicans* was detected. Among them, at 3 sites, *C*. *albicans* represented more than 80% of the bacteria tested, whereas *Streptococcus* spp. was only between 11% and 22% (Fig 2B). In 54.5% of interdental biofilms (6 from the 11 ID sites expressing *C*. *albicans*) inhabited by *C*. *albicans*, *S*. *mutans* was present. Moreover, 28% of the ID sites co-expressed *S*. *mutans* and *Lactobacillus* spp. Among them, 71.5% revealed a higher quantity of *S*. *mutans* than *Lactobacillus* spp.

**Table 3:** Distribution of the pathogens according to sites and subjects. "Positive sites" correspond to the number of sites expressing one pathogenic species or the total bacteria (TB). "Positive subjects" indicates the number of subjects expressing one pathogenic species or the total bacteria. n: total number of sites or subjects tested; *S*spp: *Streptococcus* spp.; *Sm*: *Streptococcus mutans; L*spp: *Lactobacillus* spp.; Espp: *Enterococcus* spp.; *Ef*: *Enterococcus faecalis; Ca*: Candida *albicans*.

Variable		n	*S*spp	*Sm*	*L*spp	*E*spp	*Ef*	*Ca*
**All**	**Positive sites**	100	100	28	100	99	0	11
	**Positive subjects**	25	25	11	25	25	0	7
**Age (years)**								
20–25	**Positive sites**	44	44	10	44	43	0	1
	**Positive subjects**	11	11	3	11	11	0	1
25–30	**Positive sites**	24	24	7	24	24	0	3
	**Positive subjects**	6	6	3	6	6	0	3
30–35	**Positive sites**	32	32	11	32	32	0	7
	**Positive subjects**	8	8	5	8	8	0	3
**Sex**								
Male	**Positive sites**	60	60	11	60	59	0	6
	**Positive subjects**	15	15	4	15	15	0	3
Female	**Positive sites**	40	40	17	40	40	0	5
	**Positive subjects**	10	10	7	10	10	0	4
**Arcade**								
Upper	**Positive sites**	50	50	13	50	50	0	7
	**Positive subjects**	25	25	11	25	25	0	5
Lower	**Positive sites**	50	50	15	50	49	0	4
	**Positive subjects**	25	25	13	25	25	0	4
**IDB size**								
0.6 mm	**Positive sites**	5	5	1	5	5	0	0
	**Positive subjects**	3	3	1	3	3	0	0
0.7 mm	**Positive sites**	55	55	11	55	54	0	7
	**Positive subjects**	20	20	6	20	20	0	6
0.8 mm	**Positive sites**	25	25	9	25	25	0	1
	**Positive subjects**	17	17	7	17	17	0	1
0.9 mm	**Positive sites**	8	8	3	8	8	0	2
	**Positive subjects**	5	5	2	5	5	0	1
1.1 mm	**Positive sites**	7	7	4	7	7	0	1
	**Positive subjects**	4	4	4	4	4	0	1

### Total genome count and pathogen count

**Fig 3:**
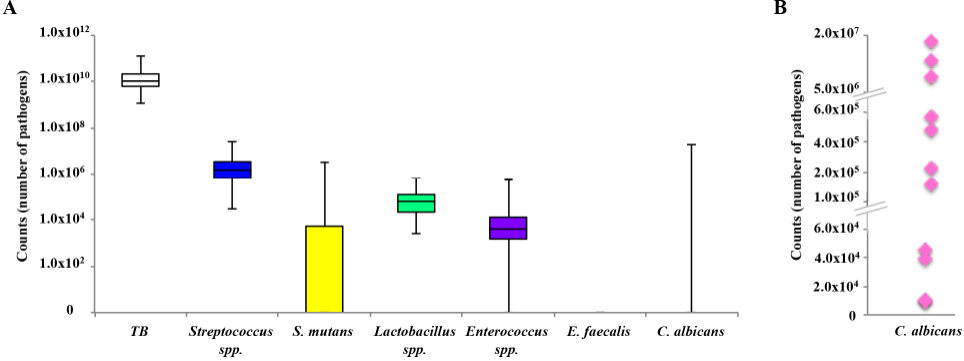
Abundance of bacterial species among the interdental sites. A. Box plots representing, for each pathogen, the first, median, and third quartiles, from bottom to top. The first box on the left corresponds to the total bacteria (TB). TB: total bacterial load. B. Count of *C*. *albicans* according to sites.

Fig 3A illustrates the abundance of the 6 evaluated pathogens in the collected samples. One interdental space (ID space) carried on average approximately 1xE10 bacteria. The pathogens tested presented various levels of expression. *Streptococcus* spp. was the most abundant species (3.2xE06 bacteria in one ID space), followed by *Lactobacillus* spp. (1.1xE05 bacteria in one ID space) and *Enterococcus* spp. (2.2xE04 bacteria in one ID space). *S*. *mutans* represented an average of 2.0xE05 bacteria in one ID space for all sites regardless of detection (Table 3). However, only in 11 of the 25 subjects tested was *S*. *mutans* detected (Table 3) with levels ranging from 3.4xE03 to 3.4xE06 bacteria in one ID space. *E*. *faecalis* was not detected. *C*. *albicans* was detected only in 11 sites (Table 3) with amounts varying from 9xE03 to 1.8xE07 bacteria in one ID space (Fig 3B).

### Impact of age and sex on the genome count

**Fig 4:**
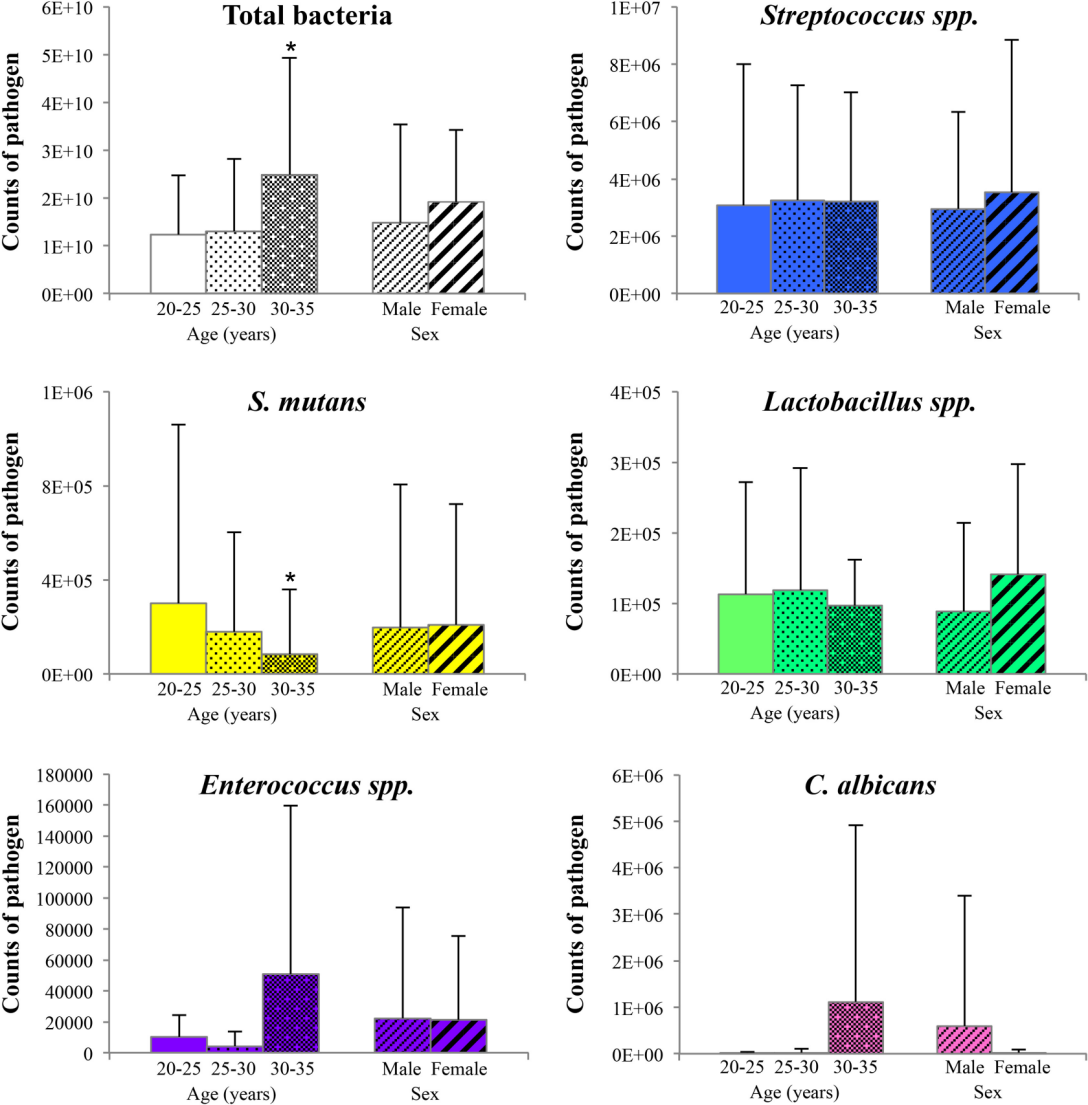
Quantification of the pathogens according to age and sex. Total counts from each pathogen were averaged across sites in each subgroup. Error bars represent standard deviations. Comparisons: * p<0.05, by using SUDAAN 7.0 (procedures DESCRIPT and REGRESS) to account for clustering (multiple sites within the subjects).

The comparison of the mean value of each pathogen according to sex and age is shown in Fig 4 and in Table 4. There was a strong increase for *C*. *albicans* (more than 200 times), for *Enterococcus* spp. (5.8 times) and a significant decrease for *S*. *mutans* (3.5 times) between the subjects aged from 20 to 25 years and those aged 30 to 35 years (p<0.05, T-test). The other pathogens tested did not appear to be affected by age. No significant differences were observed by sex.

**Table 4:** Average abundance of the 6 pathogens in various subgroups. The column labelled “TB” indicates the mean abundance of the total bacteria, whereas the other columns indicate the mean abundance of each pathogen species. Data are expressed as the mean ± standard deviation. n: number of sites; TB: total bacterial load.

Variable	n	TB	*S*spp	*Sm*	*L*spp	*E*spp	*Ef*	*Ca*
**All**	100	1.7xE10 ± 1.9xE10	3.2xE06 ± 4.2xE06	2.0xE05 ± 5.7xE05	1.1xE05 ± 1.4xE05	2.2xE4 ± 6.5xE04	0.0	3.6xE05 ± 2.2xE06
**Age (years)**								
20–25	44	1.1xE10 ± 1.1xE10	3.1xE06 ± 4.9xE06	3.0xE05 ± 7.6xE05	1.0xE05 ± 1.5xE05	1.0xE04 ± 1.4xE04	0.0	5.2xE03 ± 3.5xE04
25–30	24	1.5xE10 ± 1.7xE10	3.3xE06 ± 4.0xE06	1.8xE05 ±4.2xE05	1.4xE05 ± 1.9xE05	4.2xE03 ± 9.1xE03	0.0	2.2xE04 ± 9.1xE04
30–35	32	2.5xE10 ± 2.4xE10	3.2xE06 ± 3.8xE06	8.4xE04 ± 2.8xE05	9.7xE04 ± 6.5xE04	5.8xE04 ± 1.1xE05	0.0	1.1xE06 ± 3.8xE06
**Sex**								
Male	60	1.5xE10 ± 2.1xE10	2.9xE06 ± 3.5xE06	2.0xE05 ± 6.1xE05	8.9xE04 ± 1.3xE05	2.2xE04 ± 7.2xE04	0.0	5.9xE05 ± 2.8xE06
Female	40	1.9xE10 ± 1.5xE10	3.5xE06 ± 5.3xE06	2.1xE05 ± 5.1xE05	1.4xE05 ± 1.5xE05	2.1xE04 ± 5.4xE05	0.0	1.7xE04 ± 7.8xE04
**Arcade**								
Upper	50	1.8xE10 ± 2.3xE10	3.5xE06 ± 5.1xE06	1.9xE05 ± 5.0xE05	1.0xE05 ± 1.1xE05	3.3xE04 ± 8.9xE04	0.0	3.8xE05 ± 2.6xE06
Lower	50	1.5xE10 ± 1.4xE10	2.8xE06 ± 3.2xE06	2.2xE05 ± 6.4xE05	1.2xE05 ± 1.6xE05	1.1xE04 ± 1.7xE04	0.0	3.5xE05 ± 1.8xE06
**IDB size**								
0.6 mm	5	9.8xE09 ± 6.4xE09	1.2xE06 ± 1.1xE06	2.1xE04 ± 4.6xE04	8.9xE04 ± 3.0xE04	3.0xE03 ± 1.7xE03	0.0	0.0
0.7 mm	55	1.3xE10 ± 1.1xE10	2.1xE06 ± 2.6xE06	1.1xE05 ± 3.7xE05	9.4xE04 ± 1.3xE05	7.9xE03 ± 1.4xE04	0.0	2.5xE04 ± 1.0xE05
0.8 mm	25	1.9xE10 ± 1.9xE10	4.3xE06 ± 4.2xE06	3.3xE05 ± 8.3xE05	1.1xE05 ± 1.6xE05	3.8xE04 ± 1.1xE05	0.0	4.6xE05 ± 2.3xE06
0.9 mm	8	3.3xE10 ± 4.2xE10	2.3xE06 ± 1.6xE06	2.2xE05 ± 5.3xE05	1.1xE05 ± 6.2xE04	2.8xE04 ± 5.7xE04	0.0	2.9xE06 ± 6.4xE06
1.1 mm	7	2.6xE10 ± 2.2xE10	1.1xE07 ± 9.0xE06	5.5xE05 ± 8.7xE05	2.4xE05 ± 1.7xE05	7.8xE04 ± 1.1xE05	0.0	1.7xE04 ± 4.4xE04

### Impact of arcade location and interdental space diameter

**Fig 5:**
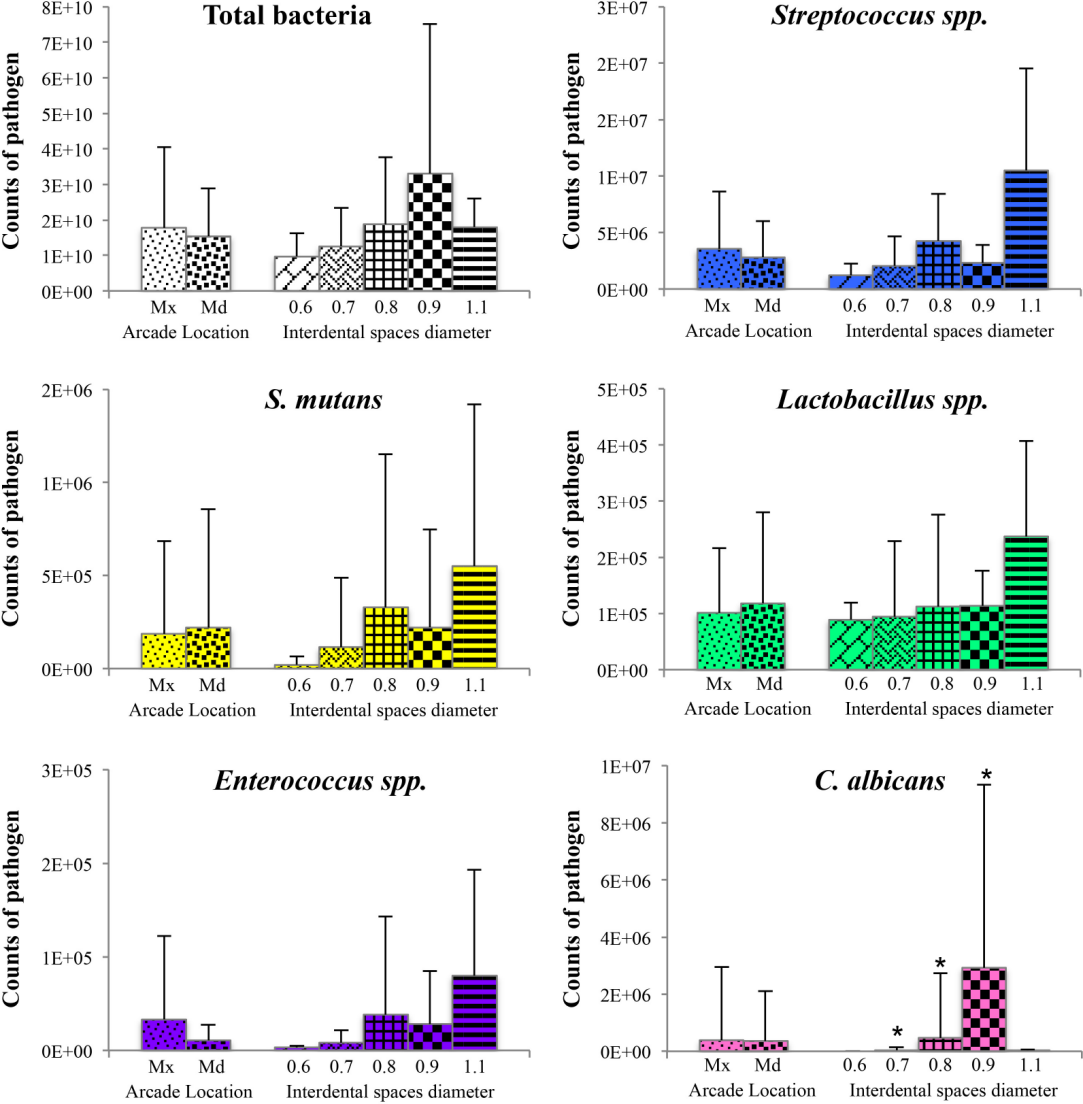
Quantification of the pathogens according to location and interdental spaces diameter. Total counts of each pathogen were averaged across sites in each subgroup. Error bars represent standard deviations. Comparisons: * p<0.05, by using SUDAAN 7.0 (procedures DESCRIPT and REGRESS) to account for clustering (multiple sites within the subjects) Mx: maxillary; Md: mandibulary.

The comparison of the mean value of each pathogen according to arcade location and the interdental space diameter is shown in Fig 5 and in Table 4. The TB and the quantity of pathogens were not significantly affected according to arcade location. The genome counts of *Streptococcus* spp., *S*. *mutans*, *Lactobacillus* spp., and *Enterococcus* spp. increased with the diameter of the interdental space except for the diameter of 0.9 mm, where the quantity was lower than for the diameter of 0.8 mm. In parallel, the number of the fungi *C*. *albicans* increased significantly for diameters ranging from 0.6 to 0.9 mm and decreased for the diameter of 1.1 mm.

### Pathogen correlations

**Fig 6:**
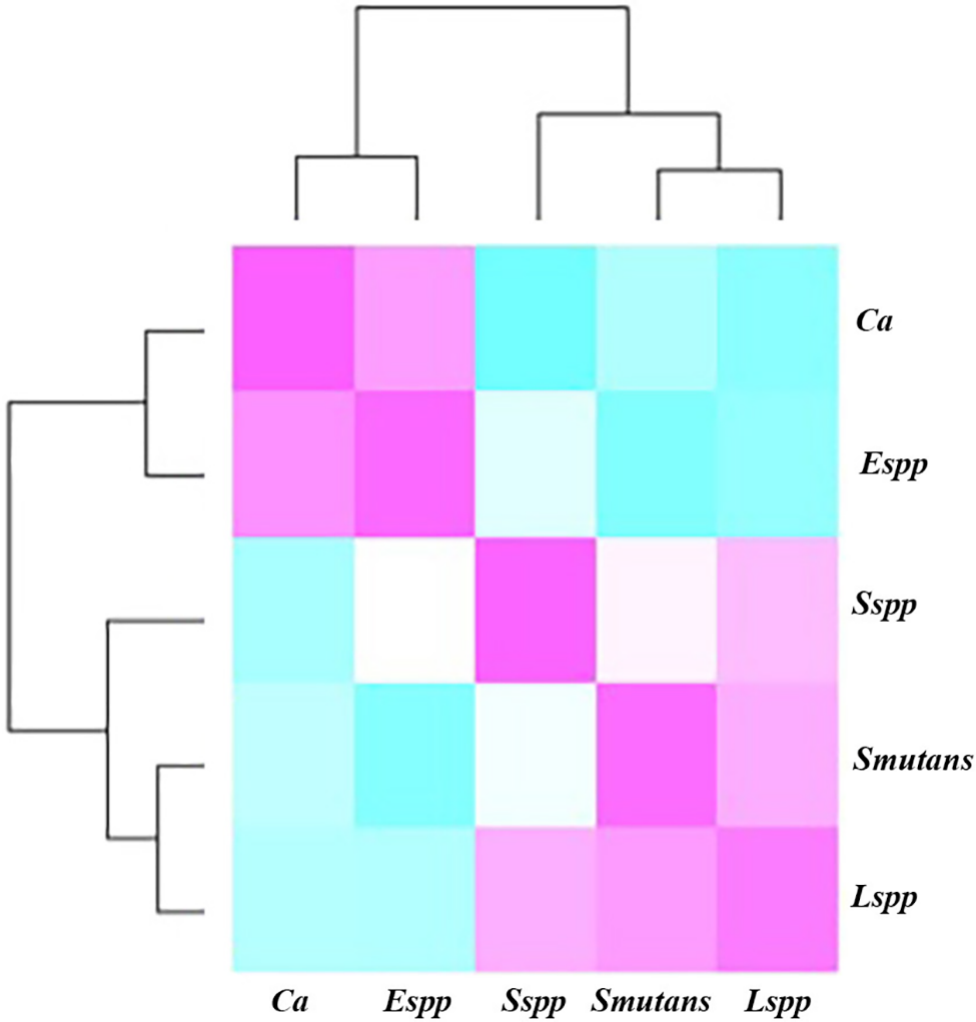
Correlation plot of the abundances of the bacterial species, corrected for age, interdental space and individual-specific effects. The pink, white, and blue squares indicate positive, zero, and negative correlations, respectively.

The dendrogram (Fig 6) underscores the correlations between our 5-pathogenic species and the 100 measured ID sites. Even after the removal of the fixed effects related to interdental space and age, and the subtraction of the inter-site correlations, the matrix still reveals a strong correlation structure, which appears as two groups (or clusters) of correlated species. The fungus *C*. *albicans* and the bacteria *Enterococcus* spp. cluster together, whereas *Streptococcus* spp., *S*. *mutans* and *Lactobacillus* spp. form one distinct cluster.

## Discussion

To the best of our knowledge, this is the first report regarding the absolute quantification of cariogenic pathogens detected in interdental biofilms from caries-free young adults. An understanding of the process associated with the initiation and progression of interproximal cariogenic diseases could be of great help in establishing effective ways to prevent this disease. In terms of oral health, the interdental space represents a very specific location. Anatomically, it is hardly accessible to brushing. Physiologically, many bacterial species are present, including virulent ones [28]. It is not only the location where periodontal diseases such as gingivitis and periodontitis are initiated but also the location of the initiation of interproximal caries.

Oral streptococci are major constituents of dental plaque [29]. They initiate the colonization process and represent more than 80% of the early biofilm constituents [30]. Their high abundance and their high prevalence (100% of ID biofilms tested were positive) suggest that they can act as a factor in the formation of oral biofilm [31].

The gender, the age and the arcade location do not impact the colonization of the ID biofilm by *Streptococcus* spp. The genus *Streptococcus* contains several species, including in particular but not exclusively *Streptococcus mutans*, *Streptococcus oralis*, *Streptococcus sanguinis*, *Streptococcus mitis*, *Streptococcus gordonii*, and *Streptococcus sobrinus*. During the carious process, these different species may play various roles [32].

Although not considered an early colonizer, the best-studied oral streptococci is the opportunistic pathogen *S*. *mutans* [33, 34]. Its prevalence in human caries cases ranges from 70 to 100% [33]. *S*. *mutans* has been linked to crown caries in children and adolescents [35, 36] and to root caries in elderly patients [37]. *S*. *mutans* was found extensively in caries-active subjects [35, 36, 38]. Its role in caries development is well established [39]. Its metabolic activity but not its concentration impacts its pathogenicity [40]. However, due to the complex interspecies interactions, there is also evidence to suggest that other species of oral streptococci may have different roles in the caries process [41].

The results demonstrate that only 28% of subjects carried *S*. *mutans*. A decrease of 3.5 times is observed between the aged subjects from 20 to 25 years and those aged from 30 to 35 years. Therefore, the older the caries-free subjects are, the lower the quantity of *S*. *mutans* detected in the ID biofilm. However, the frequency of subjects carrying *S*. *mutans* increased between the 20 to 25-year-old (27.2%) and 30 to 35-year-old (62.5%) subject groups. *S*. *mutans* could be responsible for the future carious interproximal lesions observed in adults on the distal surface of premolars [42]. Otherwise, Dani and colleagues [43] have demonstrated that the colonization of *S*. *mutans* was increased in chronic periodontitis subjects both in saliva and sub-gingival plaque samples [43]. Our previous study determined that periodontally healthy young adults carried periodontopathogenic bacteria in their ID biofilm [28]. Thus, interacting with these bacteria, *S*. *mutans* could also play a crucial role in future periodontal diseases. A change in the subject dental risk—from cariogenic to periodontopathogenic—could occur with age. This hypothesis is supported by previous results. The prevalence of periodontal diseases significantly increases in subjects older than 35 years [44]. Moreover, the microbial shift observed according to age in the supragingival biofilm and in saliva from individuals with healthy oral conditions may contribute to the initiation and prevalence of a specific oral disease according to age [12].

*Lactobacillus* spp. appear to be associated with dental carious lesions, like cariogenic bacteria, especially in the progression of caries of dentin [36, 45]. As these bacteria are unable to bind to hard, smooth surfaces, they are found in retentive zones such as pits and fissures or deep cavities. *Lactobacillus* spp. shows a high tolerance to low pH media [46].

Our study reveals that *Lactobacillus* spp. was present in all the caries-free subjects. Previous studies established a strong correlation between the *Lactobacillus* spp. counts in the oral cavity and dental caries [46]. The higher the DMFT index was, the higher the number of children harboring a high *Lactobacillus* count [47]. In some cases, they detected *Lactobacillus* spp. in the plaque of some caries-free children but at very low levels [48]. So, the fact that *Lactobacillus* spp. was detected in 100% of interdental biofilm of young caries-free subjects can be explained by (i) the higher sensitivity of the quantitative PCR compared to the culture bacteria methods [49, 50] and (ii) the age of the subjects, who are older than in other studies that focused on children.

*Lactobacillus* spp. represented 1.1xE05 bacteria in one ID space from young caries-free adults. Previously, some studies suggested a correlation between the *Lactobacillus* spp. count and caries activity, especially in children [50, 51]. Arino and colleagues [52] noticed that subjects with a *Lactobacillus* spp. level in the saliva higher than 1xE04 CFU/mL were vulnerable to caries. The absence of carious lesions in young adults with a high level of *Lactobacillus* spp. could be due to their potential suppressive effect on cariogenic microorganisms. From a review of the literature, various studies have shown that *Lactobacillus* spp. inhibits the growth of *S*. *mutans* both *in vitro* and *in vivo* [53–55]. However, contrasting findings have also been reported [56]. These variations in *Lactobacillus* colony count in different studies can be attributed to the fact that not all strains of the *Lactobacillus* family have an inhibitory effect. The *Lactobacillus* spp. exerts its anticariogenic activity in various ways [55, 57]. Moreover, the absence of signs of periodontal disease in the studied subjects could be due to the capacity of *Lactobacillus* spp. to inhibit periodontopathogens, such as *Porphyromonas gingivalis* [58].

Previous studies showed that the mutans group of *Streptococci* and the *Lactobacillus* could have a role in the induction of root surface caries [47, 59]. Interestingly, in young caries-free adults, 28% of the tested sites co-express *S*. *mutans* and *Lactobacillus* spp., and among them, 71.5% revealed a higher quantity of *S*. *mutans* than *Lactobacillus* spp. Moreover, these two-bacterial species cluster together. So, these two bacteria could be predictive markers for interproximal caries.

Another cluster of pathogens is composed of *Enterococcus* spp. and *C*. *albicans*. Enterococci may cause a variety of oral infections. Surprisingly, there is little data concerning their oral incidence and prevalence [60]. In our cohort, 99% of caries-free young adults carried *Enterococcus* spp that is higher than previously described by Sedgley and colleagues (20%) [61]. Komiyama and colleagues [62] detected Enterococci in the saliva of 14% of young adults whose periodontal and cariogenic status were not determined. Two main reasons could explain this difference. First, our study analyzed the interdental biofilm, while all other studies focused on the saliva, the lingual biofilm, or the supragingival biofilm. Second, we quantified bacterial amounts by real-time PCR and not by bacterial culture.

The quantity of *Enterococcus* spp. is lower in 30 to 35-year-old subjects than in 20 to 30-year-old subjects. This age-related difference was previously described in the saliva of subjects whose oral status was not determined [62].

To the best of our knowledge, this is the first report of arcade location variations in the oral carriage of *Enterococcus* spp. Gender does not impact the colonization of the interdental biofilm by *Enterococcus* spp. Conversely, Komiyama and colleagues [62] described that females are higher carriers than males.

Among the genus *Enterococcus*, *E*. *faecalis* is the most detected in the oral cavity [62], although it is not a common of the healthy oral flora [60, 63]. *E*. *faecalis* strains can cause serious nosocomial infections and are implicated in dental diseases as caries, periodontitis, endodontic infections, and periimplantitis [63–67].

In our study, *E*. *faecalis* was not detected, similar to previous reports that observed that the prevalence of this bacterium was lower in healthy individuals (0–20%) [68, 69] than in patients with dental diseases (up to 68%) [64, 70]. This confirms that *E*. *faecalis* is not a constituent of the oral microbiota. Further investigations are needed to determine which species of *enterococcus* are present in the interdental biofilm from caries-free adults.

Despite the fact that the key pathogens for dental caries are bacteria, previous studies have described *C*. *albicans* as greatly contributing to caries pathogenesis, particularly in children, adolescents and young adults [71, 72]. This opportunistic fungus is a common constituent of the oral biofilm [73] and can colonize surfaces of the oral cavity, such as the palate, cheek, tongue, and the hard surfaces of the teeth. As a consequence of this oral surface colonization, this fungus is also present in saliva [74].

Previous studies have demonstrated that the abundance of this yeast is a sign of high caries risk in children [75, 76]. In adults, our results showed that 28% of the subjects were carrying *C*. *albicans* in their interdental biofilm. This result is consistent with previous studies on saliva or supragingival biofilm [77, 78], in which oral carriage rates of *Candida* ranged from 5 to 75%, respectively.

Fungal colonization by *C*. *albicans* is more abundant in the ID biofilm of males than of females but is not more frequent. Moalic and colleagues [71] described contradictory results. In their study, the fungal colonization of the supragingival biofilm was more frequent in males than in females but was not more abundant. To explain our results, several hypotheses involving factors not measured in this study are conceivable: (i) the salivary flow could be decreased in females leading to a decrease in colonization [79]; (ii) low levels of pH of the male oral cavity could favor the adhesion and the proliferation of *Candida* yeast [79]; and (iii) the blood group H antigen functions as a receptor for *C*. *albicans* [80].

No significant differences were noted in the incidence of *C*. *albicans* according to age. However, the frequency of *C*. *albicans* by site was higher with age. These results complement those of Zaremba and colleagues [81], who observed that the frequency of *Candida* spp. was higher with age in a population aged 56 to 92 years. Moreover, we demonstrated that the mean number of *C*. *albicans* increases with age. In 54% of ID biofilms inhabited by *C*. *albicans*, *S*. *mutans* is present, which supports the symbiotic role of the two species [82, 83]. Numerous studies are investigating the possible role of *C*. *albicans* as a carious risk marker. However, this role seems to be called into question. Recent studies *in vitro* have suggested that *C*. *albicans* prevents caries [84, 85].

Finally, several of the studied oral pathogens are responsible for systemic diseases. *C*. *albicans* can form potentially lethal fungal masses in the heart, kidney, and brain [86, 87]. *Enterococcus* spp. and *S*. *mutans* are known to be associated with bacteremia and infective endocarditis [88, 89]. Therefore, as previously demonstrated, 34.8% of young periodontally healthy subjects with ID biofilm bled [90]. The presence of these pathogens in the ID biofilm of young adults represents a danger and must be prevented.

## Conclusions

The ID biofilm of young caries-free subjects is composed of pathogens—*Streptococcus* spp., *S*. *mutans*, *Lactobacillus* spp., *Enterococcus* spp. and *C*. *albicans*—that are able to induce interproximal caries but that are also able to act in the periodontal process. Moreover, the potential involvement of these pathogens in systemic diseases is a strong argument in favor of taking into consideration the need to disrupt the ID biofilm in oral prophylaxis.

## Supporting information

S1 Table Bacterial count for the total load of bacteria and for 6 major pathogens in the interdental biofilm. The table represents the results of 16S qPCR DNA of the healthy subjects used in this study. *Ca*: Candida albicans; IDB: Interdental Brush; *Ef*: Enterococcus faecalis; *E*spp: Enterococcus spp.; *L*spp: Lactobacillus spp.; *S*spp: Streptococcus spp.; *Sm*: Streptococcus mutans; TB: Total bacteria.
(PDF)
