# Supplementary material for: Quantification of carious pathogens in the interdental microbiota of young caries-free adults
Source: PLoS One. 2017 Oct 10;12(10):e0185804. doi: 10.1371/journal.pone.0185804 (PMC5634565; doi:10.1371/journal.pone.0185804)
Supplement: S1 Table — The table represents the results of 16S qPCR DNA of the healthy subjects used in this study. Ca: Candida albicans; IDB: Interdental Brush; Ef: Enterococcus faecalis; Espp: Enterococcus spp.; Lspp: Lactobacillus spp.; Sspp: Streptococcus spp.; Sm: Streptococcus mutans; TB: Total bacteria. (PDF) [file pone.0185804.s001.pdf]

Supplementary Table 1: Bacterial count for the total load of bacteria and for 6 major pathogens in the interdental biofilm

The table represents the results of 16S qPCR DNA of the healthy subjects used in this study. Ca: Candida albicans; IDB: Interdental Brush; Ef: Enterococcus faecalis; Espp: Enterococcus spp.; Lspp: Lactobacillus spp.; Sspp: Streptococcus spp.; Sm: Streptococcus mutans; TB: Total bacteria.

**Subject**	**Sexe 1= M; 2=F**	**Age years**	**Cadran code**	**IDB size (mm)**	**TB**	***Sspp***	***Sm***	***Lspp***	***Espp***	***Ef***	***Ca***
1	1	25	1	0.7	8,18E+09	5,41E+05	0,00E+00	5,32E+05	6,26E+03	0,00E+00	0,00E+00
1	1	25	2	0.7	6,23E+09	1,48E+06	0,00E+00	4,07E+05	2,15E+03	0,00E+00	0,00E+00
1	1	25	3	0.7	1,10E+10	1,50E+06	0,00E+00	3,86E+05	1,30E+03	0,00E+00	0,00E+00
1	1	25	4	0.8	1,08E+10	4,58E+05	0,00E+00	3,66E+05	3,40E+03	0,00E+00	0,00E+00
2	1	25	1	0.7	5,39E+09	2,33E+06	9,75E+05	1,98E+04	3,39E+03	0,00E+00	1,06E+04
2	1	25	2	0.8	6,95E+10	5,12E+06	0,00E+00	1,74E+05	4,48E+04	0,00E+00	0,00E+00
2	1	25	3	1.1	9,30E+09	9,15E+06	0,00E+00	3,44E+04	1,52E+04	0,00E+00	0,00E+00
2	1	25	4	0.8	1,64E+09	7,44E+05	2,76E+05	1,10E+04	2,44E+03	0,00E+00	0,00E+00
3	1	22	1	0.7	1,05E+10	3,71E+05	0,00E+00	1,39E+04	2,51E+03	0,00E+00	0,00E+00
3	1	22	2	0.7	1,15E+10	1,61E+06	0,00E+00	7,58E+03	2,13E+03	0,00E+00	0,00E+00
3	1	22	3	0.7	9,98E+09	4,00E+05	0,00E+00	1,17E+04	2,50E+02	0,00E+00	0,00E+00
3	1	22	4	0.7	1,07E+10	4,88E+05	0,00E+00	1,85E+04	0,00E+00	0,00E+00	0,00E+00
4	2	32	1	0.7	5,28E+09	6,93E+05	7,28E+03	4,21E+04	1,23E+04	0,00E+00	0,00E+00
4	2	32	2	0.7	1,18E+10	1,35E+06	0,00E+00	7,31E+04	1,67E+04	0,00E+00	0,00E+00
4	2	32	3	0.7	7,03E+09	2,09E+06	0,00E+00	1,22E+05	4,37E+04	0,00E+00	0,00E+00
4	2	32	4	0.7	3,96E+09	1,32E+06	0,00E+00	1,70E+05	9,53E+03	0,00E+00	0,00E+00
5	1	20	1	0.6	1,30E+10	4,43E+05	0,00E+00	9,38E+04	1,52E+03	0,00E+00	0,00E+00
5	1	20	2	0.6	1,95E+10	2,22E+06	0,00E+00	1,14E+05	4,61E+03	0,00E+00	0,00E+00
5	1	20	3	0.7	9,53E+09	8,63E+05	0,00E+00	6,99E+04	1,11E+03	0,00E+00	2,30E+05
5	1	20	4	0.6	5,80E+09	2,45E+06	0,00E+00	6,65E+04	4,97E+03	0,00E+00	0,00E+00
6	1	27	1	0.7	6,92E+09	2,75E+05	0,00E+00	6607.5	2,50E+02	0,00E+00	0,00E+00
6	1	27	2	0.8	1,19E+10	5,54E+05	0,00E+00	2,09E+04	2,50E+02	0,00E+00	0,00E+00
6	1	27	3	0.7	3,30E+09	1,18E+06	0,00E+00	4,61E+03	3,66E+03	0,00E+00	0,00E+00
6	1	27	4	0.7	5,59E+09	4,80E+05	0,00E+00	1,24E+04	1,14E+03	0,00E+00	0,00E+00
7	2	23	1	0.7	1,13E+10	3,45E+05	0,00E+00	2,81E+04	2,50E+02	0,00E+00	0,00E+00
7	2	23	2	0.7	6,71E+09	5,39E+05	0,00E+00	5,61E+04	3,61E+03	0,00E+00	0,00E+00
7	2	23	3	0.7	2,00E+10	6,62E+05	0,00E+00	1,31E+05	7,95E+03	0,00E+00	0,00E+00
7	2	23	4	0.7	1,64E+10	2,24E+05	0,00E+00	9,60E+04	2,50E+02	0,00E+00	0,00E+00
8	2	35	1	0.9	3,25E+10	5,57E+06	0,00E+00	1,44E+05	2,18E+04	0,00E+00	0,00E+00
8	2	35	2	1.1	2,44E+10	1,44E+06	1,81E+05	1,27E+05	2,13E+04	0,00E+00	0,00E+00
8	2	35	3	0.8	3,30E+10	6,20E+05	5107.5	7,65E+04	3,39E+03	0,00E+00	0,00E+00
8	2	35	4	0.9	3,20E+10	6,30E+05	3,42E+03	1,29E+05	1,02E+03	0,00E+00	0,00E+00
9	1	23	1	0.7	3,79E+10	2,27E+06	0,00E+00	1,39E+05	2,50E+02	0,00E+00	0,00E+00
9	1	23	2	0.6	3,97E+09	5,86E+05	1,03E+05	5,15E+04	1,48E+03	0,00E+00	0,00E+00
9	1	23	3	0.7	2,27E+10	1,22E+06	0,00E+00	3,62E+04	2,00E+03	0,00E+00	0,00E+00
9	1	23	4	0.8	7,27E+10	7,88E+06	3,41E+06	6,21E+05	5,17E+04	0,00E+00	0,00E+00
10	1	24	1	0.7	6,05E+09	7,16E+06	0,00E+00	7,17E+04	1,31E+04	0,00E+00	0,00E+00
10	1	24	2	0.7	6,11E+09	5,84E+06	0,00E+00	4,87E+04	6,86E+03	0,00E+00	0,00E+00
10	1	24	3	0.7	8,03E+09	3,27E+06	0,00E+00	9,08E+04	1,19E+04	0,00E+00	0,00E+00
10	1	24	4	0.7	7,50E+09	8,40E+06	0,00E+00	7,14E+04	3,30E+04	0,00E+00	0,00E+00
11	2	21	1	0.7	6,35E+09	2,88E+06	0,00E+00	2,94E+05	5,03E+04	0,00E+00	0,00E+00
11	2	21	2	0.7	5,26E+09	1,71E+06	0,00E+00	7,27E+04	4,74E+03	0,00E+00	0,00E+00
11	2	21	3	0.7	1,65E+10	1,97E+06	0,00E+00	7,27E+04	4,84E+03	0,00E+00	0,00E+00
11	2	21	4	0.8	2,02E+10	4,67E+06	0,00E+00	5,51E+05	1,48E+04	0,00E+00	0,00E+00
12	1	22	1	0.9	6,30E+09	1,51E+06	0,00E+00	6,31E+04	4,01E+03	0,00E+00	0,00E+00
12	1	22	2	0.8	6,05E+09	1,24E+06	0,00E+00	3,05E+04	9,98E+02	0,00E+00	0,00E+00
12	1	22	3	0.7	6,87E+09	8,25E+05	0,00E+00	4,04E+04	1,20E+03	0,00E+00	0,00E+00
12	1	22	4	0.9	6,15E+09	2,39E+06	0,00E+00	9,08E+03	7,80E+03	0,00E+00	0,00E+00
13	1	22	1	0.8	6,55E+09	2,68E+06	0,00E+00	1,64E+04	6,69E+03	0,00E+00	0,00E+00
13	1	22	2	0.8	7,23E+09	1,30E+07	0,00E+00	8,93E+04	3,12E+04	0,00E+00	0,00E+00
13	1	22	3	0.8	6,71E+09	2,75E+06	0,00E+00	1,95E+04	5,67E+03	0,00E+00	0,00E+00
13	1	22	4	0.8	1,18E+10	3,44E+06	0,00E+00	2,24E+04	2,88E+03	0,00E+00	0,00E+00
14	2	27	1	0.7	4,37E+10	1,44E+06	0,00E+00	3,47E+05	5,30E+03	0,00E+00	0,00E+00
14	2	27	2	0.7	2,70E+10	1,05E+06	0,00E+00	1,25E+05	4,11E+03	0,00E+00	0,00E+00
14	2	27	3	0.7	4,08E+10	1,33E+05	0,00E+00	1,22E+05	2,50E+02	0,00E+00	0,00E+00
14	2	27	4	0.7	4,33E+10	8,48E+06	0,00E+00	6,57E+05	2,50E+02	0,00E+00	4,86E+05

15	1	32	1	0.8	3,87E+10	7,58E+05	0,00E+00	3,44E+04	6,26E+04	0,00E+00	0,00E+00
15	1	32	2	0.9	1,32E+11	3,39E+06	0,00E+00	1,90E+05	1,67E+05	0,00E+00	0,00E+00
15	1	32	3	0.7	2,52E+10	1,80E+05	0,00E+00	1,01E+05	1,90E+03	0,00E+00	0,00E+00
15	1	32	4	0.8	2,39E+10	9,45E+05	0,00E+00	4,10E+04	2,51E+04	0,00E+00	0,00E+00
16	2	26	1	0.7	7,10E+09	1,28E+06	8,93E+05	6,47E+04	2,27E+03	0,00E+00	0,00E+00
16	2	26	2	0.7	1,07E+10	1,92E+06	1,73E+06	3,70E+04	2,50E+02	0,00E+00	9,00E+03
16	2	26	3	0.7	1,28E+10	8,18E+05	2,49E+05	3,20E+04	1,70E+03	0,00E+00	0,00E+00
16	2	26	4	0.7	3,92E+09	2,44E+05	1,55E+05	3,27E+04	2,50E+02	0,00E+00	0,00E+00
17	1	30	1	0.7	1,72E+10	5,36E+04	0,00E+00	2,41E+04	2,50E+02	0,00E+00	0,00E+00
17	1	30	2	0.6	6,35E+09	2,87E+05	0,00E+00	1,19E+05	2,26E+03	0,00E+00	0,00E+00
17	1	30	3	0.8	1,11E+10	5,45E+05	0,00E+00	3,41E+04	4,91E+03	0,00E+00	0,00E+00
17	1	30	4	0.9	3,67E+10	1,04E+06	0,00E+00	6,37E+04	1,28E+04	0,00E+00	0,00E+00
18	2	33	1	1.1	3,63E+10	1,19E+07	0,00E+00	2,52E+05	1,60E+05	0,00E+00	0,00E+00
18	2	33	2	1.1	7,10E+10	1,82E+07	0,00E+00	2,34E+05	3,05E+05	0,00E+00	1,17E+05
18	2	33	3	1.1	1,88E+10	3,56E+06	4,34E+05	1,27E+05	4,50E+04	0,00E+00	0,00E+00
18	2	33	4	0.8	2,19E+10	5,55E+06	0,00E+00	1,85E+04	7,44E+04	0,00E+00	0,00E+00
19	2	33	1	0.7	3,56E+10	1,05E+06	0,00E+00	2587.5	2,63E+03	0,00E+00	4,57E+04
19	2	33	2	0.7	2,30E+10	1,96E+06	1,88E+05	4,22E+04	4,15E+03	0,00E+00	3,88E+04
19	2	33	3	0.7	2,15E+10	6,05E+06	3,97E+04	4,54E+04	1,07E+03	0,00E+00	0,00E+00
19	2	33	4	0.8	3,79E+10	5,53E+06	1,57E+04	1,54E+05	2,50E+02	0,00E+00	0,00E+00
20	2	29	1	0.7	4,13E+09	1,28E+05	5,45E+03	1,01E+04	2,50E+02	0,00E+00	0,00E+00
20	2	29	2	0.7	1,17E+09	3,04E+04	0,00E+00	3472.5	2,50E+02	0,00E+00	0,00E+00
20	2	29	3	0.7	7,65E+09	8,48E+04	0,00E+00	6637.5	1,16E+03	0,00E+00	0,00E+00
20	2	29	4	0.7	3,82E+09	6,79E+05	0,00E+00	1,16E+04	1,28E+03	0,00E+00	0,00E+00
21	1	30	1	0.7	7,38E+09	2,34E+06	0,00E+00	1,25E+05	9,90E+03	0,00E+00	5,69E+05
21	1	30	2	0.9	1,26E+10	2,50E+06	1,50E+06	1,76E+05	8,48E+03	0,00E+00	1,82E+07
21	1	30	3	0.9	6,77E+09	1,44E+06	2,71E+05	1,33E+05	2,75E+03	0,00E+00	5,23E+06
21	1	30	4	0.8	1,10E+10	3,44E+06	3,89E+04	9,38E+04	1,82E+03	0,00E+00	1,14E+07
22	1	23	1	0.7	5,11E+09	8,55E+05	0,00E+00	7,20E+03	4,43E+03	0,00E+00	0,00E+00
22	1	23	2	0.7	1,72E+09	4,66E+05	0,00E+00	7,01E+03	1,55E+03	0,00E+00	0,00E+00
22	1	23	3	0.7	2,19E+09	1,55E+06	0,00E+00	4,78E+04	3,85E+03	0,00E+00	0,00E+00
22	1	23	4	0.7	3,34E+09	1,39E+06	0,00E+00	2,45E+04	4,07E+03	0,00E+00	0,00E+00
23	1	24	1	0.7	6,22E+09	1,13E+07	2,07E+05	5782.5	5,51E+04	0,00E+00	0,00E+00
23	1	24	2	0.8	7,39E+09	3,95E+06	1,04E+06	6,36E+03	2,79E+04	0,00E+00	0,00E+00
23	1	24	3	0.8	9,23E+09	1,58E+07	2,18E+06	3,11E+04	1,73E+04	0,00E+00	0,00E+00
23	1	24	4	0.7	1,01E+10	8,70E+06	1,83E+06	5,06E+04	2,63E+04	0,00E+00	0,00E+00
24	2	23	1	0.8	1,20E+10	1,25E+07	5,40E+03	7,80E+04	9,53E+03	0,00E+00	0,00E+00
24	2	23	2	1.1	7,58E+09	2,61E+07	2,40E+06	3,23E+05	7,88E+03	0,00E+00	0,00E+00
24	2	23	3	0.8	1,14E+10	2,87E+06	1,20E+06	1,79E+05	4,11E+03	0,00E+00	0,00E+00
24	2	23	4	1.1	1,17E+10	3,29E+06	8,33E+05	5,58E+05	3,57E+03	0,00E+00	0,00E+00
25	1	34	1	0.8	2,30E+10	7,17E+06	0,00E+00	9,60E+04	5,33E+05	0,00E+00	0,00E+00
25	1	34	2	0.8	2,71E+09	2,51E+06	0,00E+00	1,85E+04	1,36E+04	0,00E+00	0,00E+00
25	1	34	3	0.8	2,98E+09	2,12E+06	0,00E+00	1,87E+04	3,78E+03	0,00E+00	0,00E+00
25	1	34	4	0.7	2,24E+10	6,74E+06	0,00E+00	7,58E+04	5,39E+04	0,00E+00	0,00E+00
